# *In utero* alcohol exposure increases mammary tumorigenesis in rats

**DOI:** 10.1038/sj.bjc.6601793

**Published:** 2004-05-11

**Authors:** L Hilakivi-Clarke, A Cabanes, S de Assis, M Wang, G Khan, W J Shoemaker, R G Stevens

**Affiliations:** 1 Lombardi Cancer Center and Department of Oncology, Georgetown University, 3970 Reservoir Road, NW, Washington, DC 20007, USA; 2 Department of Psychiatry, University of Connecticut Health Center, 263 Farmington Avenue, Farmington, CT 06030-6325, USA; 3 Department of Community Medicine, University of Connecticut Health Center, 263 Farmington Avenue, Farmington, CT 06030-6325, USA

**Keywords:** *in utero* exposures, alcohol, breast cancer, oestrogen receptor, mammary gland

## Abstract

Findings in humans and animal models suggest that *in utero* hormonal and dietary exposures increase later breast cancer risk. Since alcohol intake by adult women consistently increases their breast cancer risk, we wondered whether maternal alcohol consumption during pregnancy increases female offspring's mammary tumorigenesis. In our study, pregnant female rats were pair-fed isocaloric diets containing either 0 (control), 16 or 25 g alcohol kg^−1^ feed between days 7 and 19 of gestation. These alcohol exposures generate blood alcohol levels that correspond to low and moderate alcohol consumption and are lower than those that induce foetal alcohol syndrome. Serum oestradiol levels were elevated in pregnant rats exposed to alcohol (*P*<0.003). When adult, female offspring of alcohol-exposed dams developed significantly more 7,12-dimethylbenz[a]anthracene -induced mammary tumours, compared to the controls (tumour multiplicity; mean±s.e.m., controls: 2.0±0.3, 16 g alcohol: 2.7±0.4 and 25 g alcohol: 3.7±0.4; *P*<0.006). In addition, the mammary epithelial tree of the alcohol-exposed offspring was denser (*P*<0.004) and contained more structures that are susceptible for the initiation of breast cancer (*P*<0.001). Immunohistochemical assessment indicated that the mammary glands of 22-week-old *in utero* alcohol-exposed rats contained elevated levels of oestrogen receptor-*α* (*P*<0.04) that is consistent with the changes in mammary gland morphology. In summary, maternal alcohol intake during pregnancy increases female offspring's mammary tumorigenesis, perhaps by programming the foetal mammary gland to exhibit persistent alterations in morphology and gene expression. It remains to be determined whether an increase in pregnancy oestradiol levels mediated alcohol's effects on offspring's mammary tumorigenesis.

Some breast cancers may be pre-initiated *in utero* following an exposure to elevated maternal oestrogen levels that stimulate the growth of foetal mammary gland and might cause long-lasting alterations in the expression of oestrogen-regulated genes (Trichopoulos, 1990; Hilakivi-Clarke *et al*, 2002). Our goal has been to identify factors that modify pregnancy oestrogen levels and daughter's breast cancer risk. These studies have to be performed first using animal models that mimic human breast cancer, since it is difficult to examine interactions between *in utero* exposures and breast cancer that is detected several decades later in women.

One factor that might modify pregnancy hormone levels is diet: for example, maternal intake of a high-fat n-6 polyunsaturated fatty acid diet increases both pregnancy oestradiol (E2) levels and carcinogen-induced mammary tumorigenesis among female offspring, mimicking the effects seen when pregnant dams are treated with E2 (Hilakivi-Clarke *et al*, 1997b). Another dietary factor that might modify pregnancy E2 levels is alcohol. Alcohol intake increases serum oestrogen levels (Hankinson *et al*, 1995), possibly by stimulating aromatase activity (Purohit, 2000), and alcohol has been consistently linked to increased risk of developing breast cancer (Smith-Warner *et al*, 1998; Singletary and Gapstur, 2001; Hamajima *et al*, 2002). Studies in humans indicate that mothers reporting having consumed some alcohol during pregnancy have higher pregnancy E2 levels than women who do not drink any alcohol during pregnancy (Petridou *et al*, 1992; Wuu *et al*, 2002). It is not known, however, whether alcohol exposure *in utero* through a pregnant mother increases later breast cancer risk (Stevens and Hilakivi-Clarke, 2001).

The aim of our study was to determine whether maternal alcohol exposure increases pregnancy E2 levels and female offspring's risk of developing mammary tumours in rats. In addition, we studied possible changes in the mammary gland morphology and expression of oestrogen receptor (ER)-*α*. These receptors mediate the effects of oestrogens on the breast, through a complex signalling network, and are clearly involved in the mammary gland development (Saji *et al*, 2000). Findings *in vitro* indicate that alcohol increases ER-*α* expression in human breast cancer cells (Fan *et al*, 2000; Singletary *et al*, 2001). Further, earlier human studies have shown that high alcohol intake is linked to increased mammographic density (Vachon *et al*, 2000) that in turn is associated with increased breast cancer risk (Boyd *et al*, 2001). Findings in animal studies also suggest that alcohol modifies mammary gland development (Singletary *et al*, 1991). The goal of our study was to determine whether alcohol exposure during pregnancy might increase mammary gland ER-*α* levels, epithelial density and number of epithelial targets for malignant transformation (terminal end buds (TEBs)).

## METHODS

### Maternal alcohol exposure

Pregnant Sprague–Dawley rats were obtained from Charles River on day 7 of gestation. The rats were housed individually in standard rat plexiglas cages, at a constant temperature and humidity, under a 12-h light–dark cycle (lights on 0600 h). All the experiments reported in this manuscript were carried out with the appropriate institutional ethical committee approval and they met the standards of both the US federal regulations and those required by the UKCCCR guidelines (Workman *et al*, 1998).

To examine whether maternal alcohol intake affects pregnancy oestrogen levels and offspring's mammary tumorigenesis, the rats were divided into three groups (*n*=15 per group) upon arrival on gestation day 7, and were pair-fed isocaloric liquid diets containing either 0 (control), 16 g (low, 7% alcohol of total energy) or 25 g (moderate, 15% alcohol of total energy) alcohol kg^−1^ feed (Workman *et al*, 1998). Other ingredients of the diets were Premix, oil, maltose-dextrin, and deionised water. Diet ingredients were provided by Research Diets, Inc., New Brunswick, N. J. Pre-mix contained the following ingredients (given as g l^−1^): casein (41.4), DL-methionine (0.3), L-cystine (0.5), cellulose (10), xanthan gum (3), complete mineral mix (8.75), complete vitamin mix (2.5) and choline bitartrate (0.53). The oil mix contained corn oil (8.5), olive oil (28.4) and safflower oil (2.7) The control diet also had 115.2 g of maltodextrin, and the alcohol-containing diets had an amount of maltodextrin removed equivalent to the calories of the added alcohol. Diets were prepared twice a week using a commercial blender.

We did not determine blood alcohol levels (BAL) in this experiment, but were able to estimate them based on previous studies that used identical diets used here (Vavrousek-Jakuba *et al*, 1991; Catlin *et al*, 1993; Baker and Shoemaker, 1995). Our estimates were also based on the linearity of BAL following administration of different levels of alcohol. According to these estimates, BAL for the rats receiving the 16 g alcohol kg diet is 61.3±5.0 mg dl^−1^, and for the 25 g alcohol kg diet is 95.8±6.1 mg dl^−1^. These BAL are similar to those seen in low-to-modest alcohol drinkers, respectively, and lower than those found to induce foetal alcohol syndrome (FAS) (Singletary and Gapstur, 2001).

Since alcohol intoxication might disrupt normal labour and care of newborn pups, we withdrew pregnant rats from alcohol on gestation day 19. All the animals were fed AIN93 diets from that point onwards.

### Effects on pregnancy oestrogen levels

On gestation day 19, blood was obtained by cardiac puncture to determine circulating 17*β*-oestradiol (E2) levels in dams fed 0 (control), 16 or 25 g alcohol per kg feed between gestation days 7 and 19. For that purpose, a specific double antibody kit from ICN Biomedicals, Inc. (Irvine, CA, USA) was used following the manufacturer's instructions. To avoid any cross-reactivity with other serum components, E2 was extracted from serum using a mixture of organic solvents.

### Mammary gland morphology

The mechanisms mediating the effects of *in utero* exposures on later risk of developing breast cancer could include changes in the mammary gland morphology and expression of genes regulating it. Therefore, wholemounts of the fourth abdominal glands, obtained at different ages and processed as previously described (Hilakivi-Clarke *et al*, 1997a,1997b), were assessed for changes in the morphology (Workman *et al*, 1998). The relative densities of epithelial tree, alveolar buds and lobules were determined in the 8- and 22-week-old rats using a five-point visual scale (from: 0=absent, to 5=numerous) we have previously validated (Hilakivi-Clarke *et al*, 1997a). The total number of TEBs was counted in 3-, 6-and 8-week-old offspring; that is, at times when their number is at the highest and before they differentiate to lobular–alveolar structures. Identification of each of the epithelial structures was based on the guidelines of mammary gland morphology by Russo and Russo (1987). All the analyses were done double-blinded under an Olympus dissecting microscope.

### Oestrogen receptors

We determined ER-*α* expression in the mammary glands of 22-week-old rats by immunocytochemistry using MC-20 antibody for ER-*α* (rabbit polyclonal IgG, Santa Cruz Biotechnology, CA, USA) at 1 : 100 dilution. The level of expression was assessed in three ductal and three lobular structures per section. Oestrogen receptor-*α* protein levels were quantitated by using a 0–5 scale (O=lowest and 5=highest) to determine the percentile of cells showing nuclear staining, and a 0–3 scale to determine the staining intensity. The average scores for percentile cells stained and staining intensity were added together separately in lobules and ducts.

We also investigated the validity of our quantification method. This was done by determining ER-*α* levels in 8-week-old nontreated rats that were randomly divided to three groups (*n*=5 each). If significant differences were found among the groups that should be similar (all nontreated animals), this would suggest that our approach did not reliably predict the true differences between the control and experimental mammary glands.

### Effects on offspring's mammary tumorigenesis

Mammary tumours were induced by administration of 10 mg (∼50 mg kg^−1^ body weight) 7,12-dimethylbenz(a)anthracene (DMBA) (Sigma, St Louis, MO, USA) to 47-day-old female rats (*n*=23–25 per group) (Workman *et al*, 1998). The histopathology, oestrogen dependence, oestrogen and progesterone receptor expression and antioestrogen responsiveness of the DMBA-induced tumours closely reflect human breast cancer (Russo and Russo, 1987). More than 75% of the tumours induced by 10 mg DMBA are adenocarcinomas (Russo and Russo, 1996); in our previous experiments, the proportion of adenocarcinomas in the DMBA control group was 80–100% (Hilakivi-Clarke *et al*, 2000). The carcinogen was dissolved in peanut oil and administered by oral gavage in a volume of 1 ml.

The animals were examined for mammary tumours by palpation once per week. The end points for data analysis were: (i) latency to tumour appearance, (ii) the number of animals with tumours (tumour incidence), (iii) the number of tumours per animal (tumour multiplicity) and (iv) tumour growth rate. During the follow-up, those animals in which tumour burden approximated 10% of total body weight were killed, as required by our institution. All surviving animals, including those that did not appear to develop mammary tumours, were killed 17 weeks after carcinogen administration.

### Statistical analyses

Results for the data obtained on: (i) pregnancy-related parameters (pregnancy weight gain, birth weight, number of pups per litter and pregnancy serum E2 levels); (ii) mammary gland morphology (total number of TEBs and density of epithelial tree, ABs and lobules in the whole mounts), (iii) ER-*α* protein levels and (iv) some mammary tumour end points (latency and multiplicity) were analysed using one- or two-way analysis of variance (ANOVA). When determining latency, only the week when the first tumour was detected was assessed. Animals that did not develop tumours were included in the analysis and given a latency value of the length of follow-up plus one week (18 weeks). When assessing tumour multiplicity, animals that did not develop any tumours were given a value 0. Where appropriate, between-group comparisons were done using Fisher's least significant difference (LDS) test. Differences in the tumour incidence on week 17 were determined using a *χ*^2^ test. The time to tumour presentation was measured as the number of weeks from DMBA exposure to the time the first tumour per animal could be palpated. Estimations of tumour presentation were calculated by the methods developed by Kaplan and Meier (1958). Differences among the treatment arms were tested using an extension of the log rank test and both Gehan's and Peto's generalised Wilcoxon tests as implemented in STATISTICA (Stat soft, 1998). The differences were considered significant if the *P*-value was less than 0.05. All probabilities are two-tailed.

## RESULTS

### Effects on pregnancy outcome and pregnancy E2 levels

Maternal alcohol exposure did not affect the litter size, birth weight or postnatal weight gain (data not shown). Vaginal opening of the female pups, reflecting puberty onset, was not either affected by *in utero* alcohol exposures (data not shown).

**Figure 1:**
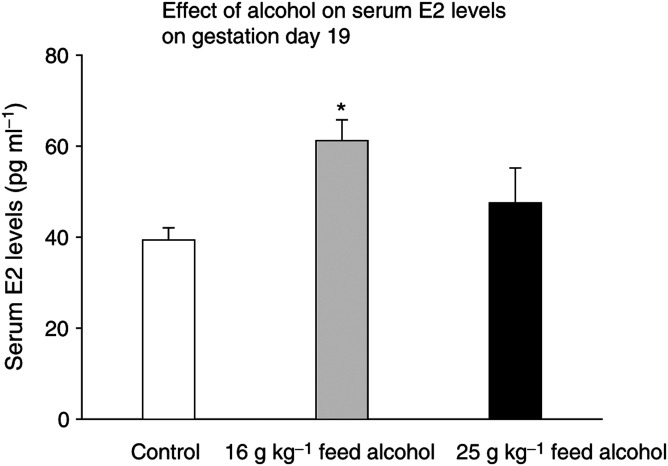
Circulating oestradiol (E2) levels on gestation day 19 in rats fed 0 g (control), 16 g or 25 g alcohol kg^−1^ feed between gestation days 7 and 19. The E2 levels were significantly higher in the animals consuming 16 g alcohol than in the controls, ^*^P<0.05.

The E2 levels were significantly higher in the pregnant dams consuming 16 g alcohol than in the controls (*P*<0.05; F(2,27)=7.3, *P*<0.003) (Figure 1

). The difference between the controls and moderate-alcohol-exposed group (25 g alcohol per kg feed) did not reach statistical significance.

### Effects on offspring's mammary tumorigenesis

**Figure 2:**
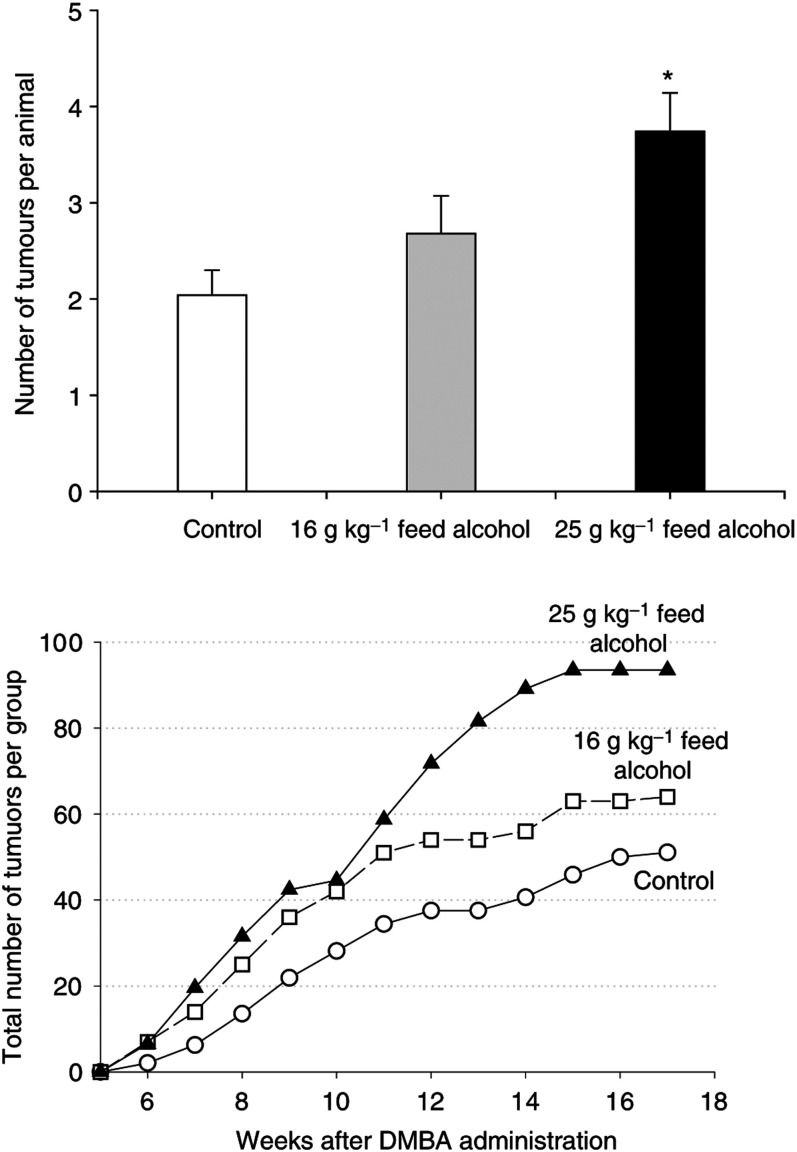
Mammary tumour multiplicity (number of tumours per animal at sacrifice) and total number of tumours per week in rats exposed *in utero* to 0 g (control, *n*=24), 16 g (*n*=25) or 25 g (*n*=23) alcohol kg^−1^ feed, following an exposure to 10 mg 7,12-dimethylbenz(a)anthracene (DMBA) on postnatal day 47. The data shown for total number of tumours are standardised to 24 animals per group, using the formula: 24 × total number of tumours per week, divided by the number of animals per group. These latter data are shown to illustrate the differences among groups, and thus were not subjected to statistical analysis. Tumour multiplicity was increased in the 25 g kg^−1^ feed alcohol-exposed rats, compared to the controls: ^*^P<0.05.

Tumour multiplicity (the total number of palpable tumours per animal) was significantly higher in the rats exposed *in utero* to alcohol than in the controls (F(2,69)=5.53, *P*<0.006) (Figure 2A and B

). *Post hoc* analysis revealed that the increase was significant between the control and moderate-alcohol groups. The tumour incidence in all groups approached 100% and, therefore, no differences in this end point among *in utero* alcohol-exposed and control rats were seen. However, the incidence was consistently higher in the alcohol groups than controls during each of the 17 weeks of tumour growth monitoring (Figure 2c).

Latency to the appearance of the first tumour was longer in the control group, though not significantly so (mean±s.e.m. weeks, control: 9.7±0.6, 16 g alcohol: 8.4±0.5, 25 g alcohol: 8.6±0.5). Latency to any subsequent tumours (control: 10.5±0.4, 16 g alcohol: 9.7±0.3, 25 g alcohol: 10.2±0.3) was not significantly different among the groups. Further, tumour growth rates were similar in all the three groups (data not shown).

### Mammary gland morphology

**Figure 3:**
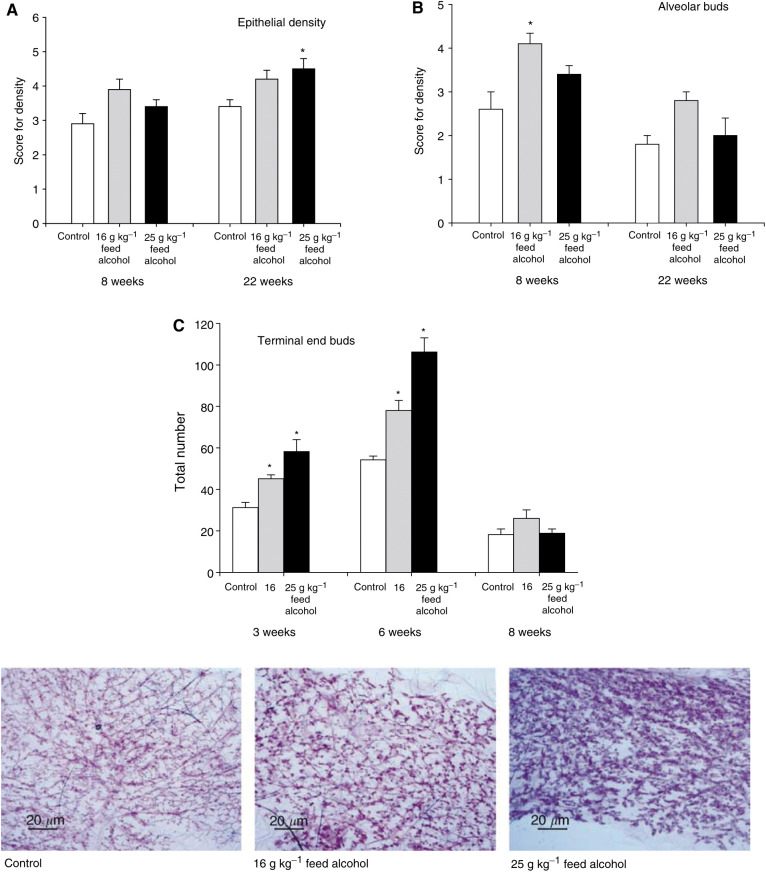
*In utero* exposure to 0 g (control), 16 g or 25 g alcohol kg^−1^ feed increase in (**A**) epithelial density and (**B**) the density of alveolar buds, determined in 8- and 22-week-old rats. No changes were noted in the density of lobules. The total number of (**C**) TEBs, assessed in wholemounts of 3-, 6- and 8-week-old rats, was also dose-dependently increased. Statistically different from the controls: ^*^P<0.05. Representative wholemounts of the fourth abdominal gland in 22-week-old offspring are shown.

*In utero* exposure to alcohol increased epithelial density (F(2,24)=7.21, *P*<0.004) and the density of alveolar buds (F(2,24)=8.88, *P*<0.001), determined in 8- and 22-week-old rats (Figures 3a and b

). No changes were noted in the density of lobules (data not shown). The total number of TEBs, assessed in wholemounts of 3-, 6- and 8-week-old rats, was dose-dependently increased during weeks 6 and 8 (F(2,39)=12.6, *P*<0.001) (Figure 3c). Representative wholemounts of the fourth abdominal gland in 22-week-old offspring of dams exposed to control, low (16 g kg^−1^ feed) and moderate (25 g kg^−1^ feed) alcohol during pregnancy are also shown (Figure 3d). Our findings indicate that *in utero* alcohol exposure increases mammary epithelial density and the number of targets for malignant transformation.

#### Oestrogen receptor-α

The validation study that determined ER-*α* expression using immunohistochemistry in the mammary glands of three similar groups of nontreated animals showed no differences in either the lobular or ductal ER-*α* expression among the groups. The proportion of cells stained and staining intensity in the nontreated animals in three lobular and three ductal structures per gland were evaluated using scales of 0–5 and 0–3, respectively. The combined scores in the lobules of the three nontreated groups were 3.3±0.2, 3.4±0.2 and 3.8±0.2, and in the ducts 3.7±0.3, 3.2±0.3 and 3.2±0.2. These results suggest that our quantitative approach for immunohistochemical assays is unlikely to yield false-positive results.

**Figure 4:**
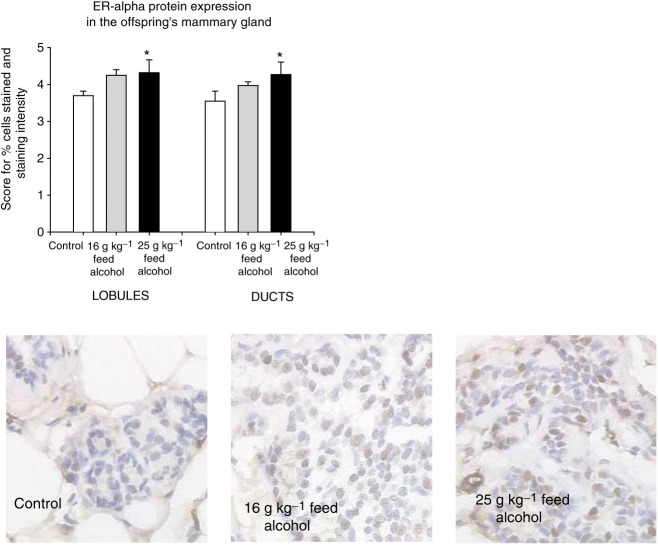
*In utero* exposure to 0 g (control), 16 g or 25 g alcohol kg^−1^ feed increased oestrogen receptor (ER)-*α* levels in the mammary glands of 22-week-old rats. Statistically different from the controls: ^*^P<0.05. Representative sections of each of the three treatment groups for ER-*α* staining are shown.

We then determined ER-*α* expression in the mammary glands of 22-week-old rats exposed *in utero* to 0, 16 or 25 g alcohol kg^−1^ feed. The results showed that the levels of ER-*α* protein were elevated in the mammary gland of the rats exposed to alcohol *in utero* (ER-*α* (F(2,18)=3.9, *P*<0.04) (Figures 4a and b

). These changes were dose-dependent and significant only in the offspring of dams consuming 25 g alcohol kg^−1^ feed-containing diets during pregnancy, when compared to the controls.

## DISCUSSION

Many dietary factors are suggested to be linked to either increased or reduced breast cancer risk, but the data supporting these associations are mostly contradictory. Alcohol consumption is an exception, and there is a consensus that alcohol intake increases the risk of developing breast cancer (Smith-Warner *et al*, 1998; Singletary and Gapstur, 2001; Hamajima *et al*, 2002). Some epidemiological studies suggest that alcohol drinking during adolescence and early adulthood has the strongest impact on breast cancer risk (Harvey *et al*, 1987; Hiatt *et al*, 1988; Young, 1989; Garland *et al*, 1999), while results of some studies indicate that drinking later in life increases breast cancer risk most (Longnecker *et al*, 1995; Swanson *et al*, 1997). Data obtained in the present study suggest that alcohol exposure already *in utero* through a pregnant mother may increase breast cancer risk.

The idea that some breast cancers are pre-initiated *in utero* is supported by several indirect observations in human populations. These studies show that women who were exposed to high foetal oestrogenic environment; that is, had a high birth weight or are twins, are at an increased risk of developing breast cancer (Braun *et al*, 1995; Michels *et al*, 1996; Sanderson *et al*, 1996; Weiss *et al*, 1997). At a particularly high risk are twins whose birth weight was high (Hubinette *et al*, 2001). A recent study also found that women exposed to the synthetic oestrogen diethylstilbestrol (DES) *in utero* are at an increased risk of developing breast cancer (Palmer *et al*, 2002). Animal studies support the hypothesis. In rats, maternal exposure to either oestradiol or DES increases the offspring's breast cancer risk (Walker, 1984; Hilakivi-Clarke *et al*, 1997b).

We found that maternal exposure to relatively low alcohol levels increased pregnancy E2 levels in rats. Previous human studies have shown that both acute and chronic alcohol consumption increases circulating E2 levels in non-pregnant women (Hankinson *et al*, 1995), possibly by alcohol-induced increase in aromatase activity (Purohit, 2000). A positive association between alcohol intake during pregnancy and pregnancy oestrogen levels in humans has also been reported (Petridou *et al*, 1992; Wuu *et al*, 2002). Thus, the present data in animals and earlier data in humans show that maternal alcohol consumption increases pregnancy E2 levels. However, since the dams kept on the highest alcohol diet did not show a significant increase in pregnancy E2 levels, although this group showed most differences in tumorigenesis, it remains to be determined whether the increased oestrogenicity in alcohol-exposed pregnant dams explains their offspring's increased mammary tumorigenesis.

The mechanisms mediating the effect of *in utero* alcohol exposure on mammary tumorigenesis may be linked to changes in mammary gland morphology and gene expression. Oestrogens stimulate normal mammary epithelial and tumour cell proliferation (Nandi *et al*, 1995), also when administered neonatally (Hilakivi-Clarke *et al*, 1997a,1997b). *In vitro* and *in vivo* studies show that alcohol increases normal and malignant mammary cell proliferation (Singletary *et al*, 2001). In humans, alcohol is associated with increased mammographic density (Vachon *et al*, 2000), and increased density is linked to four- to six-fold increase in breast cancer risk (Boyd *et al*, 2001). These findings suggest that elevated oestrogen levels *in utero* may have led to increased mammary epithelial density seen in rats exposed to alcohol *in utero*, and the increased density in turn increased the risk of developing mammary tumours.

Earlier studies have reported that rats exposed to diets containing 15–20% of energy from alcohol as juveniles exhibit an increased number of TEBs and reduced lobular and alveolar bud structures in their mammary glands (Singletary *et al*, 1991). We found that rats exposed to alcohol *in utero* had an increased number of TEBs, suggesting that alcohol exposure during foetal life modifies the pathways controlling development of the mammary gland. Further, since TEBs are targets for malignant transformation (Russo and Russo, 1987), their increased presence could explain why alcohol increases mammary tumorigenesis.

In addition to affecting mammary gland morphology, *in utero* alcohol exposure was found to increase the expression of ER-*α* in the mammary gland, consistent with earlier *in vitro* data (Fan *et al*, 2000; Singletary *et al*, 2001) and data showing that the level of alcohol intake correlates with ER-*α* expression in human breast tumours (Enger *et al*, 1999). Activation of ER-*α* induces proliferation of normal and malignant breast cells, and is associated with promotion of breast cancer (Tonetti and Jordan, 1999). If ER-*α* is important in mediating the effects of *in utero* alcohol exposure in increasing breast cancer risk, its levels should be elevated in women who develop breast cancer. This assumption is supported by findings showing that normal cells in the breasts containing a tumour express higher levels of ER-*α* than the cells in the breast containing a benign lesion (Khan *et al*, 1998). Further, breast ER-*α* levels are significantly lower in the Asian women exhibiting a low breast cancer risk than in the Caucasian women exhibiting a high breast cancer risk (Lawson *et al*, 1999).

Findings obtained in this study showing that maternal alcohol intake increased offspring's mammary tumorigenesis suggest that the role of maternal diet in modifying pregnancy oestrogen levels and affecting daughter's breast cancer risk should be investigated further. Since oestrogen levels vary by four- to six-fold among women undergoing normal pregnancy, and the reasons for the variability remain poorly understood, it is essential to identify dietary factors that modify pregnancy oestrogen levels and could therefore potentially affect daughter's breast cancer risk. Alcohol might be one of these factors. It has been known for some time that maternal alcohol consumption during pregnancy can cause FAS or its less severe manifestation, foetal alcohol effects in the offspring. The fact that maternal alcohol intake at levels that are considerable lower than those inducing FAS might also increase daughter's breast cancer risk gives further weight to the recommendation that women should avoid consuming alcohol during pregnancy.
